# Bibliographical—The Pathogenesis of Hæmorrhage

**Published:** 1869-09-15

**Authors:** Ch. Bouchard

**Affiliations:** Paris Savy. Ed.


### *Bibliographical—The Pathogenesis of Hæmorrhages.*

BY CH. BOUCHARD (PARIS SAVY. ED.).

Etiology may be deduced from pure observation; pathogenesis demands a knowledge of anatomy and physiology.

What we can accomplish to-day in this direction, corresponds exactly to the degree to which our age has arrived in its notions of normal anatomy and physiology, and it is thus that pathogenesis may become positive.

Medicine formerly could not attain to such pathogenesis; in what relates to hæmorrhage M. Bouchard proves it, in his historical sketch, in which is shown, in a striking manner, the outflow of imagination which served Stahl as his pathological physiology.

The circulatory system is an aggregation of canals closed every where; how can the blood escape, by transudation or by rupture?

The ancients believed, by the former method, diapedesis. M. Bouchard very formally rejects this. The issue by exosmosis from the vessels of the coloring matter of the blood dissolved in the serum, must not be confounded with hæmorrhage. The recent and curious experiments of M. Cohnheim, according to which the white globules of the blood escaped from the vessels by means of amœboid movements, and the red globules by pre-existing stomata, are not yet beyond the limits of controversy, and already it has been demonstrated that the pretended stomata are only illusions of the microscopist.

Hence normal blood, anatomically and chemically complete, escapes from the vessels by rupture only.

Rupture, moreover, either by exaggeration of the tension of the blood, or by diminution of external pressure and of the support which the surrounding tissues impart to the vessels, or by alteration in the consistence of the vessels themselves.

The first process is much the most frequent. Rupture, moreover, in the small vessels is much more frequent in the little veins than in the arteries; pathological anatomy furnishes proof of the fact.

The author gives a theoretical explanation of this. As regards the true capillaries without perivascular sheathes, they perhaps never rupture. Physiologically, the arterioles and especially the veins are little disposed to rupture, in consequence of their extreme dilatability, which, moreover, pertains to them by their situation and their office in the circulation.

It is in these that the paralytic congestions depending upon the nervous system (fever), are manifested; here, also, is accumulated increased arterial pressure (plethora) or venous (obstruction of the veins.)

In the second manner those hæmorrhages occur which take place upon the surface of cavities suddenly emptied, of which the capacity is suddenly increased, especially if the vessels are unsupported except by an adventitious membrane, (hæmorrhagic pleurisy and pericarditis), the hæmorrhages from compression in persons who work in the midst of compressed air, cerebral, renal, splenic and pulmonary hæmorrhages, those of soft morbid tissues, certain other conditions of the state of the vessels and of the circulatory functions being given. The author enunciates without discussion the doctrine of M. Feltz upon infarctus; infarctus would involve always the tearing of the vessels and the infiltration of the blood, the opinion which we should desire to see prevail over that of Lefeuver (Thesis, Paris, 1867, No. 195), according to whom infarctus is sufficiently characterized by the mortification of a tissue deprived of its nu ritive elements by arrest of arterial circulation.

